# Genome-wide analysis of NDR1/HIN1-like genes in pepper (*Capsicum annuum* L.) and functional characterization of *CaNHL4* under biotic and abiotic stresses

**DOI:** 10.1038/s41438-020-0318-0

**Published:** 2020-06-01

**Authors:** Changyun Liu, Haoran Peng, Xinyu Li, Chaolong Liu, Xing Lv, Xuefeng Wei, Aihong Zou, Jian Zhang, Guangjin Fan, Guanhua Ma, Lisong Ma, Xianchao Sun

**Affiliations:** 1 Laboratory of plant immunity and ecological control of plant disease, College of Plant Protection, Southwest University, 400716 Chongqing, China; 2 Department of Botany and Plant Biology, Section of Biology, Faculty of Science, University of Geneva, 1211 Geneva 4, Switzerland; 3 State Key Laboratory of North China Crop Improvement and Regulation, College of Plant Protection, Hebei Agricultural University, 071001 Baoding, China

**Keywords:** Plant immunity, Biotic

## Abstract

Plant NDR1/HIN1-like (*NHL*) genes play an important role in triggering plant defenses in response to biotic stresses. In this study, we performed a genome-wide identification of the *NHL* genes in pepper (*Capsicum annuum* L.) and characterized the functional roles of these *CaNHL* genes in response to abiotic stresses and infection by different pathogens. Phylogenetic analysis revealed that *CaNHL*s can be classified into five distinct subgroups, with each group containing generic and specific motifs. Regulatory element analysis showed that the majority of the promoter regions of the identified *CaNHL*s contain jasmonic acid (JA)-responsive and salicylic acid (SA)-responsive elements, and transcriptomic analysis revealed that *CaNHL* genes are expressed in all the examined tissues of pepper. The *CaNHL1*, *CaNHL4*, *CaNHL6*, *CaNHL10*, *CaNHL11*, and *CaNHL12* genes were significantly upregulated under abiotic stress as well as in response to different pathogens, such as TMV, *Phytophthora capsici* and *Pseudomonas syringae*. In addition, we found that CaNHL4 localizes to the plasma membrane. *CaNHL4*-silenced pepper plants display significantly increased susceptibility to TMV, *Phytophthora capsici* and *Pseudomonas syringae*, exhibiting reduced expression of JA-related and SA-related genes and reduced ROS production. However, transient overexpression of *CaNHL4* in pepper increases the expression of JA-related and SA-related genes, enhances the accumulation of ROS, and inhibits the infection of these three pathogens. Collectively, for the first time, we identified the *NHL* genes in pepper and demonstrated that *CaNHL4* is involved in the production of ROS and that it also regulates the expression of JA-related and SA-related genes in response to different pathogens, suggesting that members of the CaNHL family play an essential role in the disease resistance of pepper.

## Introduction

In nature, plants face constant attacks from insects and a diverse array of pathogens, such as bacteria, fungi, and viruses. However, plants have evolved sophisticated defense mechanisms to resist infection by these attackers. Plants have developed a two-tiered pathogen-recognition system in which they employ either membrane-localized pattern-recognition receptor proteins or cytosolic nucleotide-binding leucine-rich repeat (NB-LRR) receptor proteins to sense extracellular or intracellular immunogenic compounds secreted by pathogens. Once perceived, these compounds trigger plant defense responses against various pathogens^1–3^. Pathogen resistance mediated by resistance proteins, especially NB-LRRs, is usually associated with the hypersensitive response (HR), which is characterized by rapid programmed cell death (PCD) to limit the spread of the pathogen from the infection site^4,5^. The accumulation of reactive oxygen species (ROS) and nitric oxide (NO) at the pathogen infection site is considered important to initiate the HR, and other features, such as cell wall fortification, transcriptional reprogramming, and ion flux, are often observed^5,6^. Activation of the HR often induces systemic acquired resistance, which often relies on the salicylic acid (SA)-signaling pathway and the production of pathogenesis-related proteins^5^.

NDR1/HIN1-like (*NHL*) genes include Harpin-induced gene 1 (*HIN1*) and Nonrace-specific disease resistance gene 1 (*NDR1*) of *Arabidopsis thaliana*^7^*. HIN1* is induced by harpin protein and plays an important role in various plant defense responses, growth, development, and resistance to abiotic stresses^8^. *NDR1* has been cloned from *A. thaliana* and has been found to function in several plant disease resistance responses^7^. Most NHL proteins contain a conserved late embryogenesis abundant (LEA) domain^9^, which belongs to a protein family whose members are largely related to osmotic regulation in different organisms. Previous studies have shown that the genes encoding proteins belonging to this protein family are highly expressed during the late embryonic development of seeds as well as under environmental stresses, such as drought and low temperature^10^.

It has been documented that members of the NHL family play an important role in plant disease resistance^9^. Maldonado et al. found that overexpression of *GmNHL1* and *GmNHL8* in *A. thaliana* plants enhanced resistance to *Heterodera glycines* by activating the jasmonic acid (JA) and ethylene (ET) pathways, suggesting that *NHL1* and *NHL8* contribute to the plant defense response against pathogens^11^. Chen et al. reported that overexpression of *StPOTHR1*, a member of the *NHL* gene family, enhances resistance against *Phytophthora infestans* by restricting rapid pathogen proliferation^12^. Overexpression of *VvNHL1* (NHL 1) of *Vitis vinifera* L. in the *Arabidopsis ndr1* mutant resulted in increased resistance of the transgenic plants to *Botrytis cinerea* by enhancing cell necrosis^13^. The expression of *NHL10* in *A. thaliana* significantly increases after treatment with the cucumber mosaic virus (CMV), suggesting that this gene may play a role in plant resistance^14^. In addition, the expression of *AtNHL3* and *AtNHL25* genes from *A. thaliana* is highly induced in response to pathogen infection, and *AtNHL3* also responds rapidly to the derived signals of *Pseudomonas syringae* to generate a defense response^15^. Collectively, these findings strongly indicate that NHL proteins identified from different plant species are involved in triggering responses to a variety of biotic stresses and play an active role in inducing plant defense responses. However, the identification of *NHL* genes from pepper (*C. annuum* L.) and the functional characterization of the role of *NHL* genes in *C. annuum* disease resistance remain largely unknown. In this study, we aimed to identify the *NHL* genes in pepper in a genome-wide manner and understand the role of NHLs in pepper disease resistance, which will aid us in understanding the disease resistance of pepper.

## Results

### Identification of CaNHL genes in *C. annuum*

To identify the *NHL* genes in *C. annuum*, the sequence of the *HIN1* gene from *C. annuum* was queried against the *C. annuum* genome via BLAST. The results of the BLAST search were then refined by removing the redundant sequences and through confirmation of the presence of the LEA-2 domain using SMART. Fifteen *CaNHL* genes were obtained, which were named *CaNHL1*–*CaNHL15* based on the location within the reference genome (Table 1). To determine the chemical properties of these predicted CaNHL proteins, ProtParam tool (https://web.expasy.org/protparam/) was used to calculate the molecular weight (MW), isoelectric point (pI), and chemical formula. Interestingly, we found that the MW of the vast majority of CaNHL protein ranges from 2000 to 3000 Da, with the exception of that of CaNHL12. The pI of all CaNHL proteins is between 9 and 10. In addition, the predicted three-dimensional structure of each protein is shown in Supporting Information 1.

**Table 1:** List of identified *CaNHL* genes in *C. annuum.*

Name	Gene locus ID	Chr	Location	CDS (bp)	MW (Da)	pI	Formula
*CaNHL1*	Capana00g003650	1	63597266–63598081	816	29,899.83	9.71	C_1342_H_2167_N_363_O_385_S_11_
*CaNHL2*	Capana02g000080	2	93168389–93169156	768	29,232.24	9.98	C_1302_H_2114_N_374_O_361_S_14_
*CaNHL3*	Capana02g002134	2	150126019–150126702	684	25,936.06	9.77	C_1169_H_1830_N_328_O_319_S_11_
*CaNHL4*	Capana02g002298	2	152229022–152229801	780	29,408.50	9.63	C_1320_H_2106_N_366_O_360_S_17_
*CaNHL5*	Capana03g001817	3	220067007–220067648	642	23,985.79	9.35	C_1104_H_1714_N_278_O_307_S_6_
*CaNHL6*	Capana03g001432	3	227321791–227322579	789	29,461.79	9.95	C_1325_H_2165_N_367_O_363_S_13_
*CaNHL7*	Capana04g002564	4	10211724–10212401	678	25,569.28	9.97	C_1149_H_1788_N_330_O_320_S_7_
*CaNHL8*	Capana06g002149	6	158109052–158109642	591	22,370.18	9.89	C_1016_H_1632_N_276_O_279_S_6_
*CaNHL9*	Capana06g000641	6	227434383–227435015	633	23,626.44	9.22	C_1084_H_1685_N_283_O_295_S_7_
*CaNHL10*	Capana10g002083	10	222022991–222023674	684	26,003.05	9.09	C_1167_H_1819_N_317_O_329_S_14_
*CaNHL11*	Capana10g002082	10	222083709–222084392	684	25,999.04	9.13	C_1169_H_1823_N_317_O_329_S_13_
*CaNHL12*	Capana10g002081	10	222108128–222108451	324	12,528.35	9.27	C_556_H_878_N_158_O_162_S_5_
*CaNHL13*	Capana10g002080	10	222113163–222113798	636	23,953.74	9.67	C_1095_H_1702_N_298_O_295_S_6_
*CaNHL14*	Capana11g000361	11	248346066–248346710	645	23,881.81	9.06	C_1098_H_1726_N_284_O_301_S_5_
*CaNHL15*	Capana11g000183	11	254198867–254199487	621	23,494.29	9.90	C_1072_H_1677_N_297_O_286_S_6_

### Phylogenetic analysis of NHL proteins within the Solanaceae

**Fig. 1:**
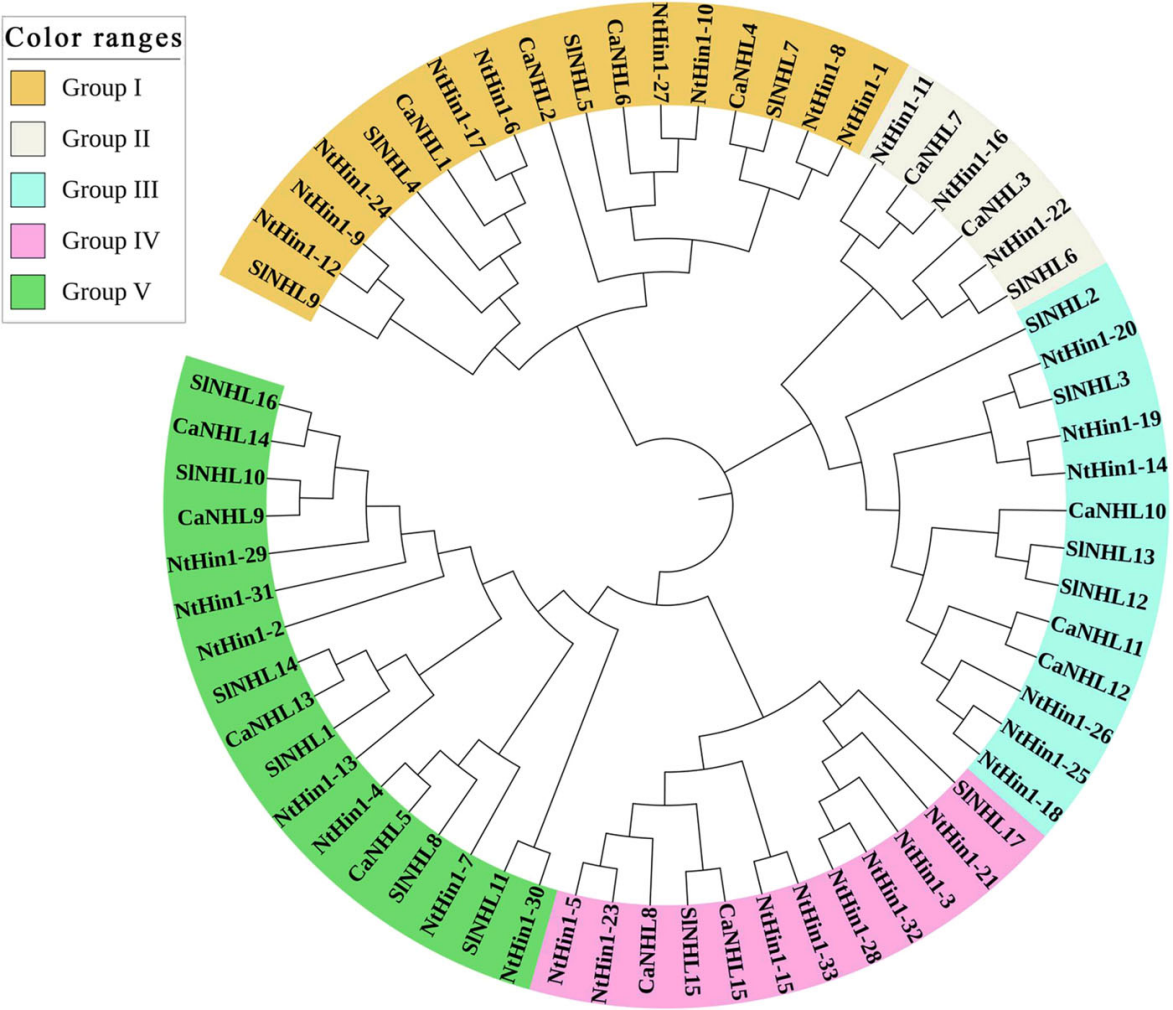
Unrooted phylogenetic tree of NHL protein family from C. annuum, S. lycopersicon, and N. tabacum MEGA 7.0.26 was used to construct thephylogenetic tree based on the NHL protein sequences, and the five distinct subgroups of NHL proteins are shown. iTOL was used to annotate and review the phylogenic tree.

To determine the evolutionary relationship among NHL proteins in *Solanaceae* species, we first retrieved the NHL protein sequences of *Solanum lycopersicon* (SlNHL) and *Nicotiana tabacum* (NtNHL) from the Sol Genomics network (https://solgenomics.net/). An unrooted phylogenetic tree was subsequently constructed using the sequence alignment data of the 15 CaNHL proteins, 15 SlNHL proteins, and 35 NtHIN1 proteins. As shown in Fig. 1, the 65 NHL proteins could be classified into five distinct groups. Group I includes CaNHL1, CaNHL2, CaNHL4, and CaNHL6; group II includes CaNHL3 and CaNHL7; group III includes CaNHL10, CaNHL11, and CaNHL12; group IV includes CaNHL8 and CaNHL15; and group V includes CaNHL5, CaNHL9, CaNHL13, and CaNHL14 (Fig. 1).

### Identification of regulatory elements and putative motifs in CaNHL proteins

**Fig. 2:**
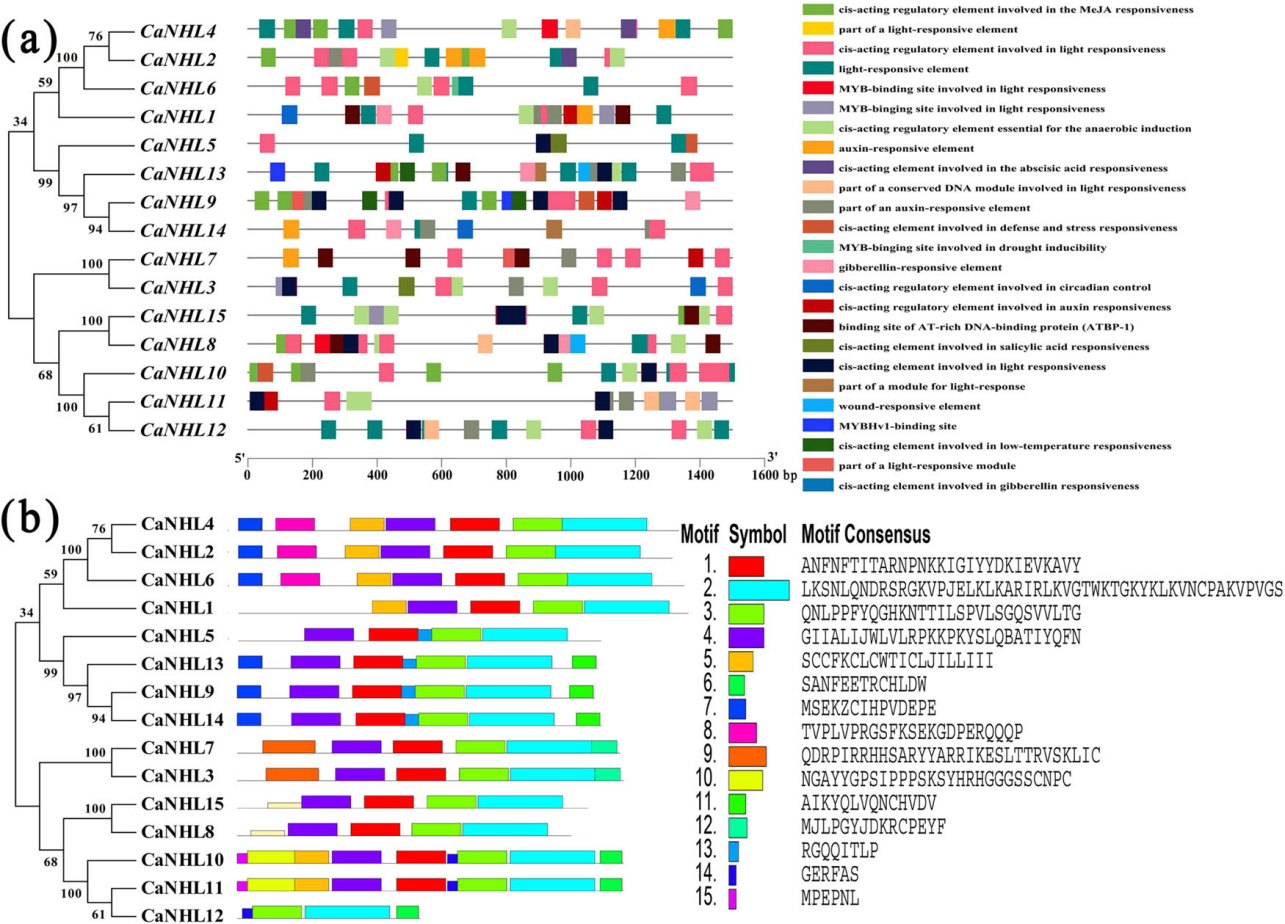
Schematic representations of the predicted regulatory elements and the conserved motifs in the CaNHL family. **a** Schematic illustration of the predicted regulatory elements in the promoter regions of CaNHLs by PlantCare. Each regulatory element is marked with a different color. **b** Schematic representation of the conserved motifs predicted in the CaNHL proteins. MEME was used to predict the conserved motifs, and TBtools was used to display the results.

The promoter region of these *CaNHL* genes was predicted by TSSP (http://www.softberry.com/berry.phtml?topic=tssp&group=programs&subgroup=promoter), and the regulatory elements in the promoter regions were predicted using information within the PlantCare database (http://bioinformatics.psb.ugent.be/webtools/plantcare/html/). The PlantCare-based analysis revealed that more than 26 regulatory elements are distributed within the promoters of the *CaNHL* genes. These elements include light-responsive elements, defense response elements and hormone induction elements, such as those for abscisic acid (ABA), auxin (IAA), gibberellin (GA), and SA (Fig. 2a). In addition, we found that the promoters of *CaNHL2*, *CaNHL4*, *CaNHL7*, *CaNHL8*, *CaNHL9*, and *CaNHL10* contain many methyl jasmonate (MeJA)-responsive elements, suggesting that these genes might be involved in the JA-signaling pathway. Thus, these observations strongly suggest that the role of NHL proteins in pepper is likely associated with disease resistance. MEME motif analysis revealed that motifs 2 and 3 are widely distributed in all CaNHL proteins (Fig. 2b). Interestingly, we found that CaNHL members within the same phylogenetic group share a similar motif composition. For example, motif 1, motif 2, motif 3, and motif 4 are present within CaNHL8 and CaNHL15, which are located on the same branch (Fig. 2b). The similar motif arrangements among the CaNHL proteins indicate that the protein architecture is conserved within specific subfamilies. However, the functions of most of these conserved motifs remain to be elucidated. Overall, our results suggest that CaNHL proteins in the same phylogenic group share conserved motifs and similar protein domain compositions, suggesting that they have similar functions in plant development and disease resistance.

### Genome-wide transcriptomic analysis of CaNHL genes and quantification of CaNHL genes under exogenous MeSA and MeJA treatments

**Fig. 3:**
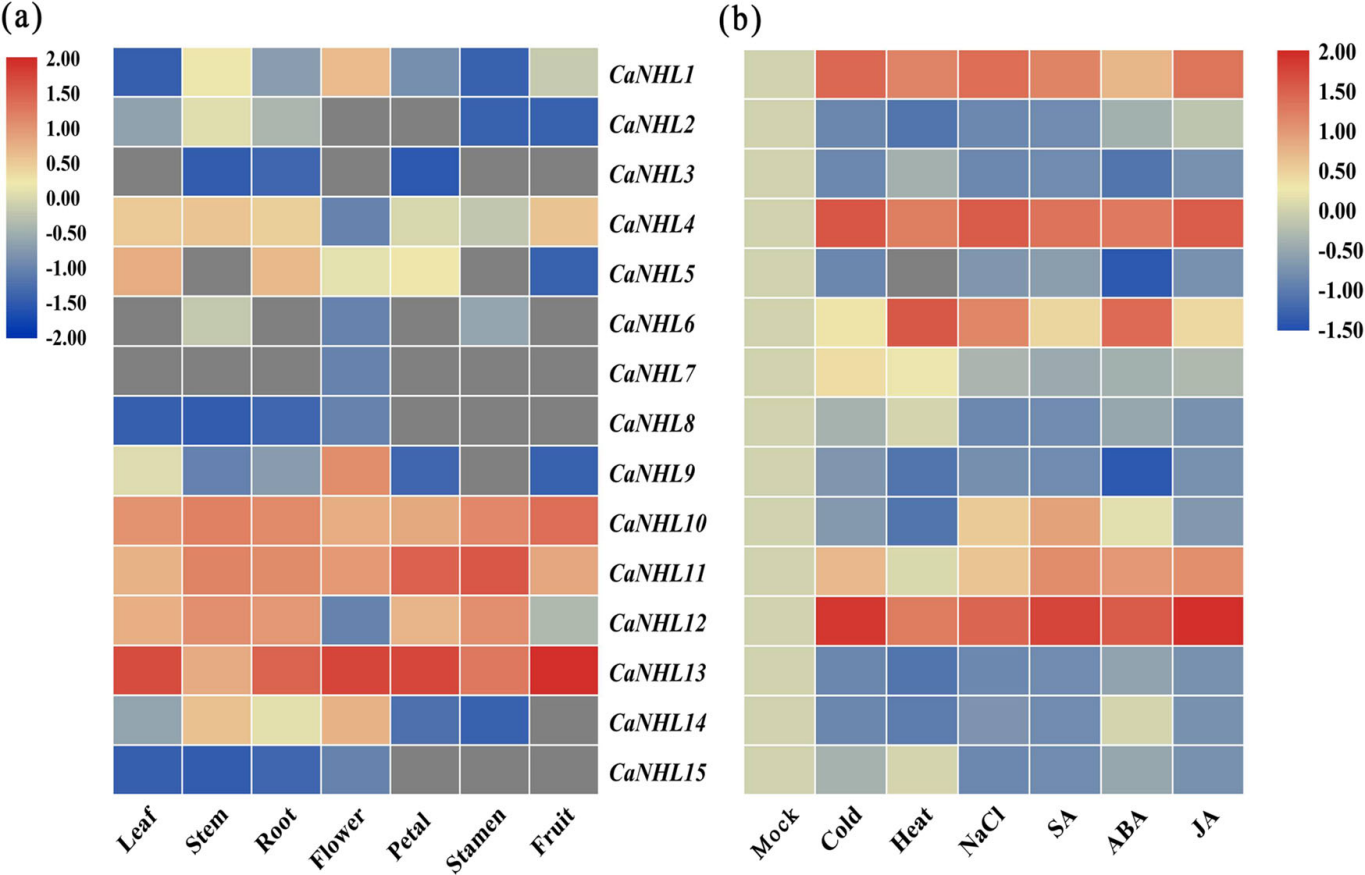
Heat map showing differential expression of CaNHL genes based on an RNA-seq transcriptomic analysis. **a** Expression of *CaNHL* genes in different pepper tissues. The columns represent the leaves, stems, roots, flowers, petals, stamens, and fruits, from left to right. **b** Expression profiles of *CaNHL* genes in response to various stresses. From left to right, the columns indicate the mock; abiotic stresses such as cold, heat, and NaCl; and hormone treatments such as SA, ABA, and JA.

To determine the global expression profiles of all *CaNHL* genes in different plant tissues and in response to various stresses, we analyzed the published RNA-seq data available in the Pepper Informatics Hub (http://pepperhub.hzau.edu.cn/) database and generated a heat map of all the *CaNHL* genes (Fig. 3). Figure 3a shows that *CaNHL10*, *CaNHL11*, and *CaNHL13* displayed constitutive expression in all examined plant tissues, including the leaves, stems, roots, flowers, petals, stamens, and fruits. However, some *CaNHL* genes exhibited tissue-specific expression patterns, and some did not show any expression. *CaNHL9* is expressed specifically in the flowers, and the expression of *CaNHL3*, *CaNHL6*, *CaNHL7*, and *CaNHL8* was not detected in any tested plant tissues. When pepper plants were challenged with different abiotic stresses, such as cold and heat, and subjected to NaCl, SA, ABA, and JA treatments, differential expression of *CaNHL* genes was observed (Fig. 3b). *CaNHL1*, *CaNHL4*, and *CaNHL12* were highly upregulated in response to all the stresses, and *CaNHL6* was specifically and highly induced in the plants subjected to heat and ABA treatments, which suggests that some of the *CaNHL* genes are involved in abiotic stress responses.

**Fig. 4:**
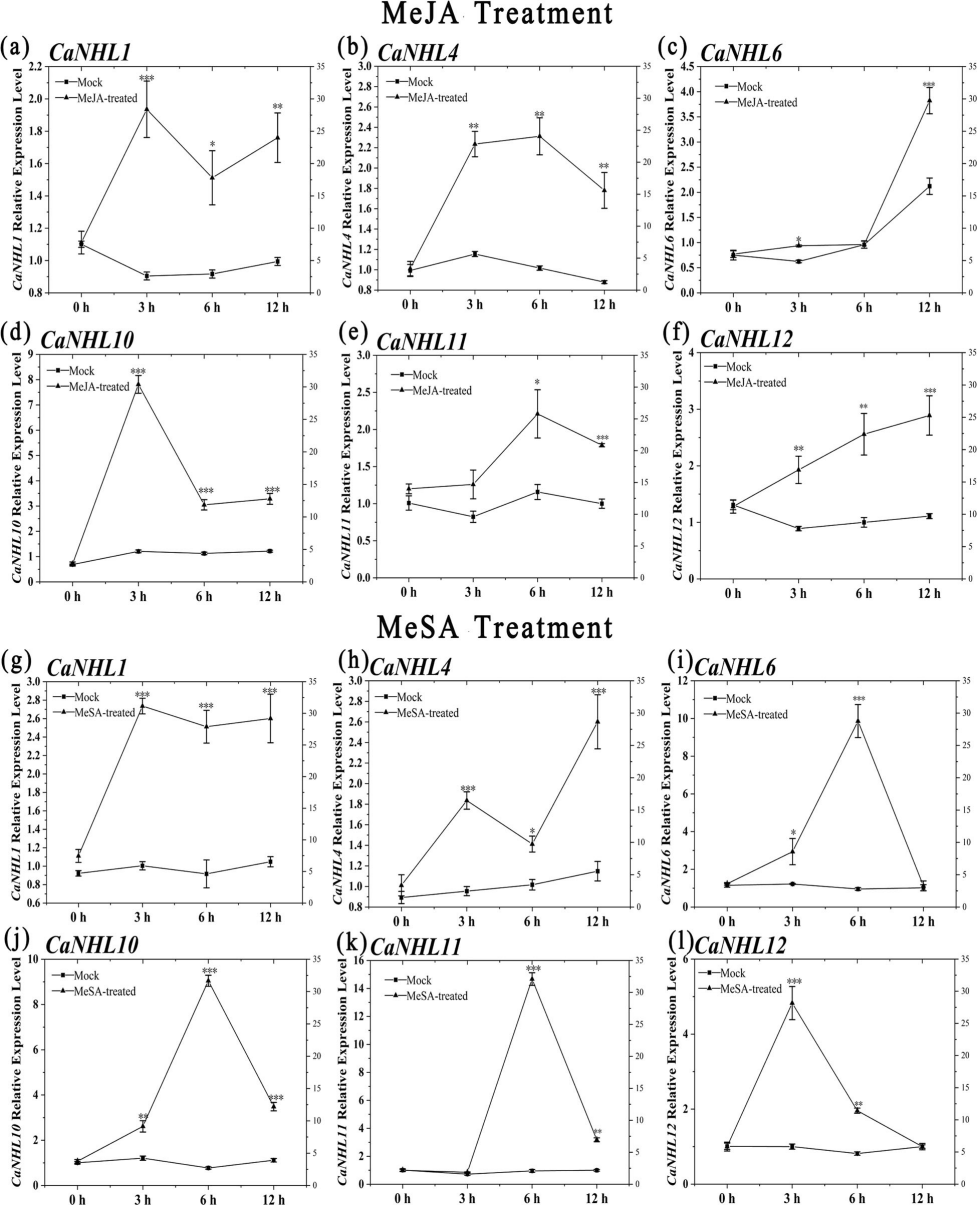
Relative expression of the *CaNHL1, CaNHL4, CaNHL6, CaNHL10, CaNHL11*, and *CaNHL12* genes in pepper leaves after MeJA and MeSA treatments. **a**–**f** Expression level of *CaNHL1*, *CaNHL4*, *CaNHL6, CaNHL10*, *CaNHL11*, and *CaNHL12* at 3, 6, 12 h after MeJA treatment. **g**–**l** Expression level of the above-mentioned *CaNHL* genes at the 3, 6, 12 h time points after MeSA treatment. The values represent the means ± standard errors (SEs) of three biological replications. The statistical analyses were performed using Student’s *t*-test (∗0.01 < *P* < 0.05; ∗∗0.001 < *P* < 0.01; ∗∗∗*P* < 0.001). “Mock” indicates plants without MeJA or MeSA treatment.

To validate the results obtained from the transcriptomic data, qPCR analysis was employed to quantify the differentially expressed *CaNHL* genes at 0, 3, 6, and 12 h post-exogenous MeJA and methyl salicylate (MeSA) treatment. MeSA is a derivative of SA, and the accumulation of MeJA and MeSA coincides with the accumulation of endogenous JA and SA^16–18^. Figure 4 shows that the expression of *CaNHL1*, *CaNHL4*, *CaNHL6*, *CaNHL10*, *CaNHL11*, and *CaNHL12* is highly induced after MeJA or MeSA treatment, which is consistent with the RNA-seq data. However, the expression pattern of individual *CaNHL* genes at specific time points after treatment is different. After MeJA treatment, the expression of *CaNHL1* increased and peaked at the 3 h time point, decreased at 6 h, and then increased again at the 12 h time point. The expression of *CaNHL4*, *CaNHL10*, and *CaNHL11* increased but then decreased, while the expression of *CaNHL6* and *CaNHL12* continued to increase and peaked at the 12 h time point after treatment (Fig. 4). After the application of MeSA, the expression of *CaNHL4* increased at the 3 h time point, after which it decreased but then peaked at the 12 h time point. The expression of the remaining five genes increased first, peaked at the 3 or 6 h time point and then decreased at the 12 h time point. Taken together, our findings demonstrate that six *CaNHL* genes are highly activated by MeSA and MeJA treatments.

### Expression of *CaNHL* genes in response to *P. capsici*, *P. syringae*, and TMV infection

**Fig. 5:**
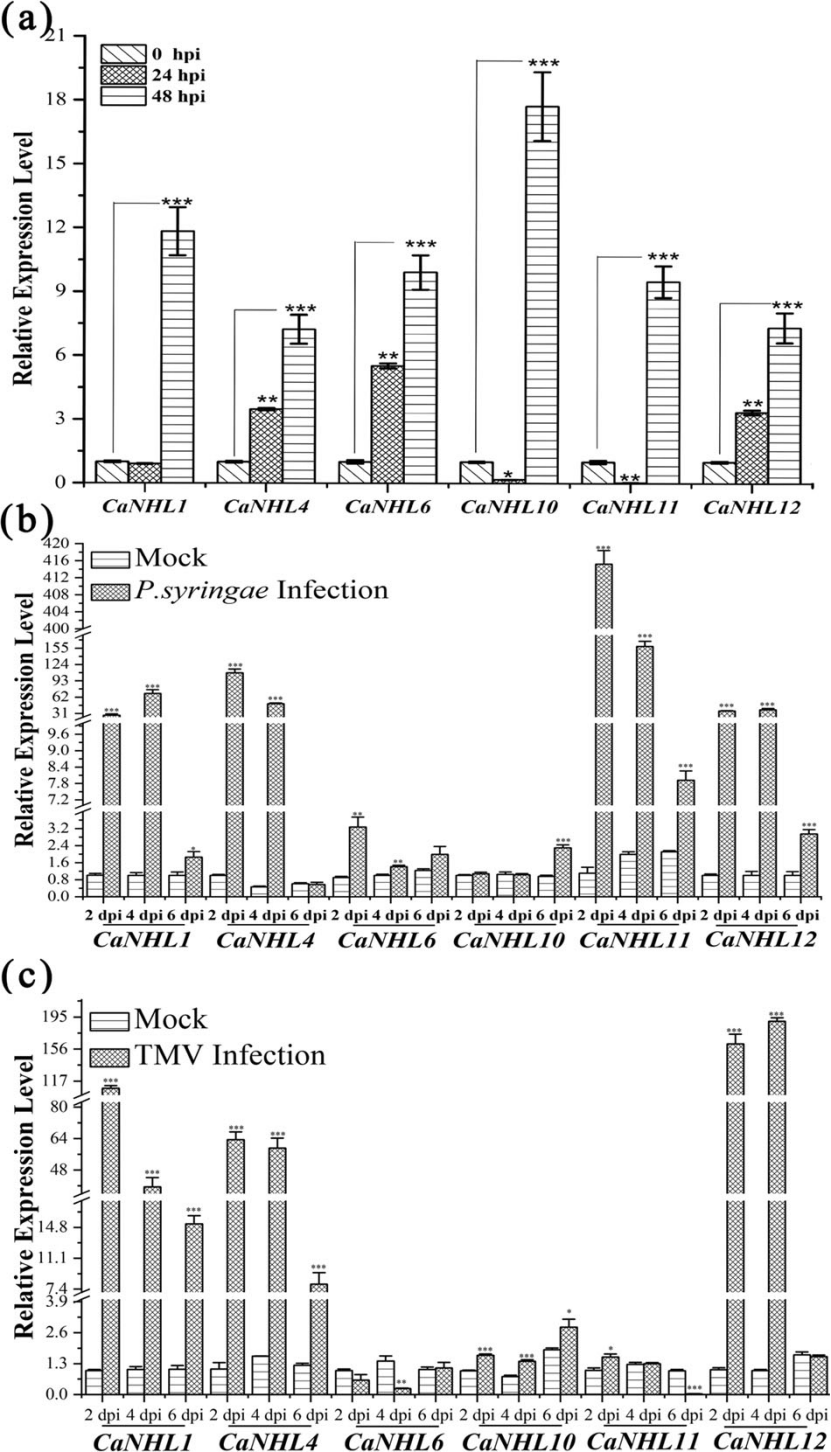
Relative expression of the *CaNHL1, CaNHL4, CaNHL6, CaNHL10, CaNHL11*, and *CaNHL12* genes post-pathogen inoculation. **a** Expression of *CaNHL1*, *CaNHL4*, *CaNHL6*, *CaNHL10*, *CaNHL11*, and *CaNHL12* post-*P. capsici* infection. **b** Expression of *CaNHL1*, *CaNHL4*, *CaNHL6*, *CaNHL10*, *CaNHL11*, and *CaNHL12* under *P. syringae* infection. **c** Expression of *CaNHL1*, *CaNHL4*, *CaNHL6*, *CaNHL10*, *CaNHL11*, and *CaNHL12* under TMV infection. The values represent the means ± standard errors (SEs) of three biological replications. The statistical analyses were performed using Student’s *t*-test (∗0.01 < *P* < 0.05; ∗∗0.001 < *P* < 0.01; ∗∗∗*P* < 0.001). “Mock” indicates plants without pathogen inoculation.

To further assess the expression profiles of *CaNHL* genes in response to biotic stresses, we inoculated pepper plants with *P. capsici*, *P. syringae*, and TMV. Total RNA was extracted from samples collected at 0, 24, and 48 h post-*P. capsici* inoculation and at 2, 4, and 6 d after *P. syringae* and TMV inoculation. As shown in Fig. 5a, the *CaNHL4*, *CaNHL6*, and *CaNHL12* genes were significantly induced at 24 hpi with *P. capsici*, while the expression patterns of *CaNHL1*, *CaNHL10*, and *CaNHL11* were similar to those of the mock plants. Interestingly, at 48 hpi, the expression level of all six genes was significantly higher than that in the mock plants (Fig. 5a). After *P. syringae* inoculation, the *CaNHL1*, *CaNHL4*, *CaNHL11*, and *CaNHL12* genes were significantly induced at 2, 4, and 6 dpi in comparison to those in the mock plants, while the expression of *CaNHL10* was similar to that in the mock plants at 2 and 4 dpi (Fig. 5b). Figure 5c shows that after TMV inoculation, the expression of the *CaNHL* genes, especially *CaNHL1*, *CaNHL4*, *CaNHL10*, and *CaNHL12*, was significantly induced at 2 dpi; however, the expression of *CaNHL6* was not induced. At 4 and 6 dpi, the expression level of most genes tended to decrease, among which *CaNHL6* and *CaNHL11* showed lower expression than did those in the mock plants. Overall, these findings strongly indicated that the *CaNHL1*, *CaNHL4*, and *CaNHL12* genes are highly induced in response to infection by these three pathogens, suggesting that they might play a significant role in the defense response against them.

### Silencing CaNHL4 in pepper increases susceptibility to different pathogens

**Fig. 6:**
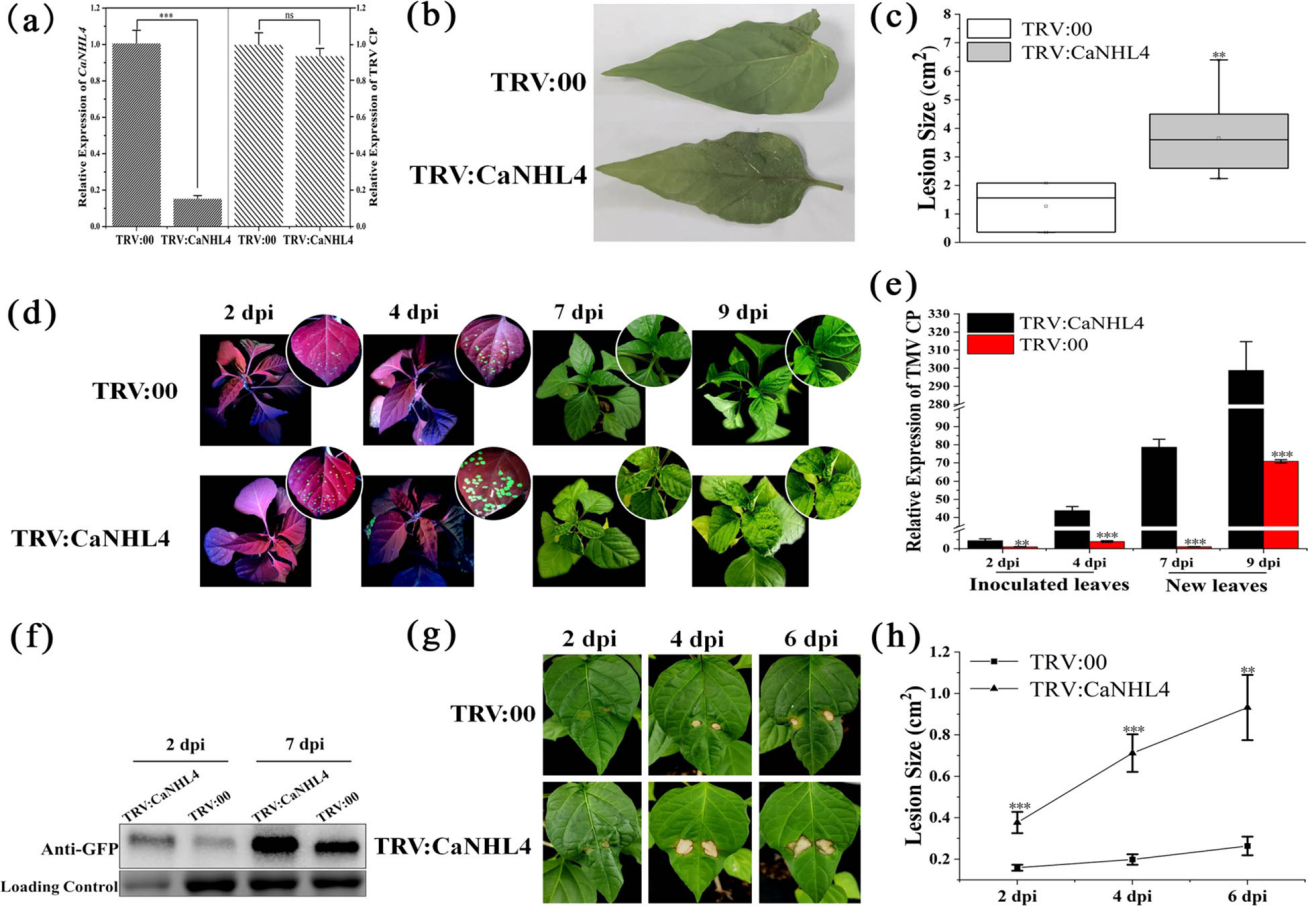
Silencing CaNHL4 in pepper enhances the susceptibility to *P. capsici*, TMV and *P. syringae*. **a** The expression level of *CaNHL4* and TRV CP was quantified by qPCR at 14 days after inoculation with TRV1 + TRV:CaNHL4 or TRV:00. **b** and **c** Silencing *CaNHL4* enhances the infection of *P. capsici*. Disease lesion size was measured and is presented. **d**–**f** Silencing *CaNHL4* promotes the movement and accumulation of TMV-GFP. The expression of TMV-CP and accumulation of TMV-GFP were measured. **g**, **h** Silencing *CaNHL4* promotes the infection of *P. syringae*. Lesion size was measured and is presented. All the experiments were repeated three times and showed similar results. The values represent means ± standard errors (SEs) of three biological replications. The statistical analyses were performed using Student’s *t*-test (∗0.01 < *P* < 0.05; ∗∗0.001 < *P* < 0.01; ∗∗∗*P* < 0.001)

As *CaNHL4* is highly induced in pepper plants subjected to MeSA and MeJA treatments and in response to pathogen infection, we decided to silence *CaNHL4* to examine the effects of *CaNHL4* silencing in pepper upon pathogen infection. We utilized the virus-induced gene silencing (VIGS) approach to silence *CaNHL4* (TRV:CaNHL4), and TRV:00 served as a mock. To determine the silencing efficacy of our designed constructs, the number of *CaNHL4* and TRV-CP transcripts was quantified by qPCR at 14 d after TRV inoculation. Figure 6a shows that the expression level of *CaNHL4* was significantly decreased relative to that of the mock plant, and no significant difference was observed in the accumulation of TRV-CP transcripts, indicating that the silencing efficacy of *CaNHL4* was sufficient for our subsequent experiments. We then simultaneously inoculated the silenced plants and mock plants with *P. capsici*, and disease development was examined at 2 dpi. As expected, larger disease lesions were observed on the silenced plants than on the mock plants, indicating that silencing *CaNHL4* enhances *P. capsici* infection (Fig. 6b, c). In addition, we inoculated the silenced plants and mock plants with TMV:GFP. Figure 6d shows that pronounced GFP signals were observed on the leaves of the silenced plants compared to the leaves of the mock plants at 2 and 4 dpi after TMV inoculation. At 7 and 9 dpi, the mosaic disease symptoms observed on the young leaves of the silenced plants developed rapidly compared to those on the mock plants. Furthermore, the number of TMV:GFP transcripts and accumulation of TRV-GFP proteins in inoculated and young leaves of the silenced plants and mock plants were quantified by qPCR and western blots, respectively. Figure 6e, f show that the expression level of GFP and the accumulation of TRV-GFP proteins in the silenced plants were significantly higher than those in the mock plants. These findings suggested that silencing *CaNHL4* enhances the infection and movement of TMV:GFP. Similarly, we inoculated the silenced plants and mock plants with *P. syringae*; the disease development was monitored, and lesion size was measured at 2, 4, and 6 dpi. Figure 6g shows that the development of the disease symptoms on the leaves of the silenced plants was significantly faster than that of the mock plants, and the lesion size on the silenced plants was significantly larger than that of the mock plants (Fig. 6h), suggesting that silencing *CaNHL4* increases the infection of *P. syringae*. Taken together, our findings reveal that silencing *CaNHL4* significantly enhances the susceptibility of pepper to infection by the three pathogens, suggesting that *CaNHL4* participates in the defense response against pathogens.

### Overexpression of *CaNHL4* in pepper enhances resistance to different pathogens

**Fig. 7:**
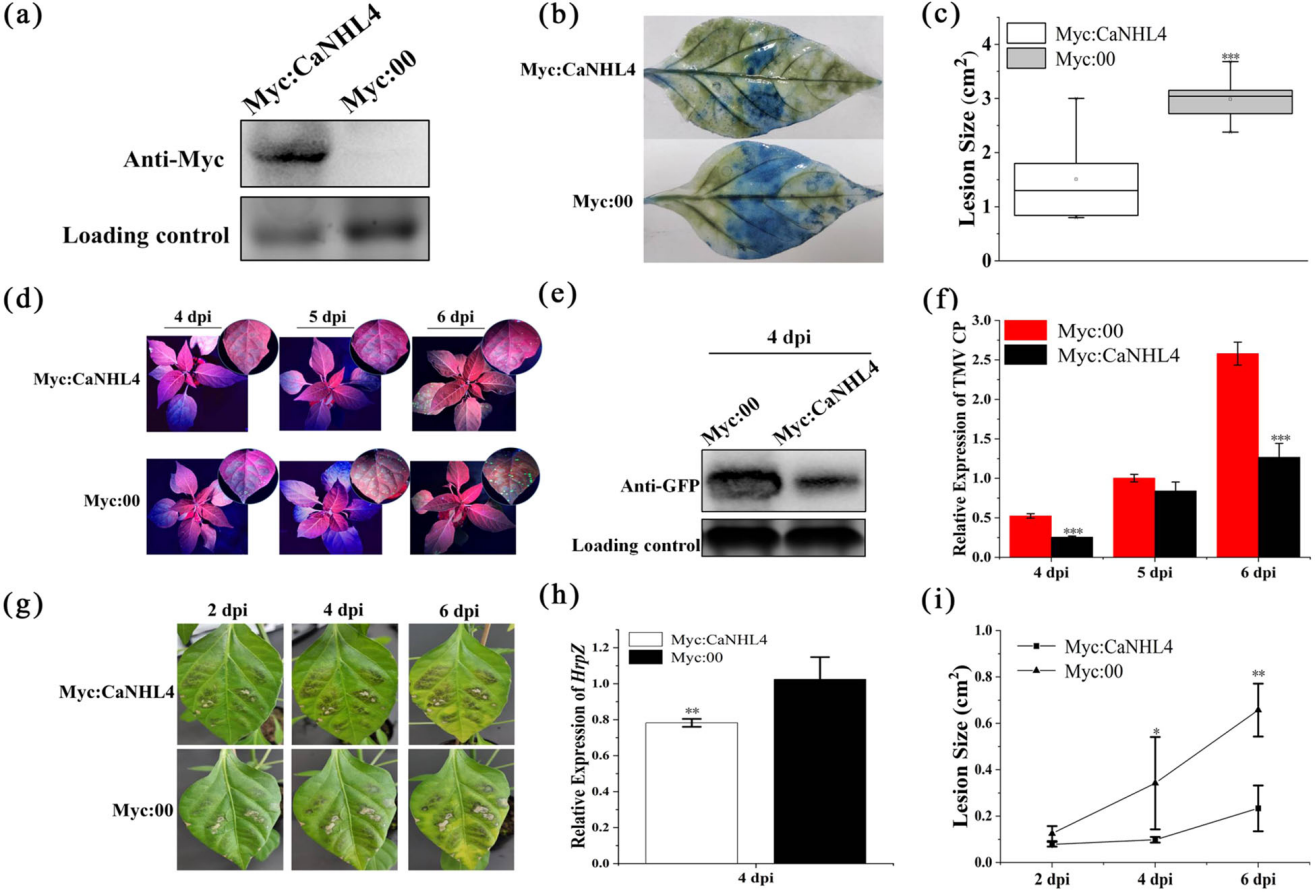
Transient overexpression CaNHL4 in pepper enhances resistance to *P. capsici*, TMV, and *P. syringae*. **a** The accumulation of Myc-CaNHL4 was quantified by western blotting at 2 d after agroinfiltration. A Myc:00 empty vector was used as a mock. **b** and **c** Transient overexpression of *CaNHL4* decreases the infection of *P. capsici*. Disease lesions were stained with trypan blue and measured. **d**–**f** Transient overexpression of *CaNHL4* inhibits the infection of TMV-GFP. The accumulation of TMV-GFP in *CaNHL4* overexpressing leaves was reduced. The number of TMV CP transcripts significantly decreased, and the accumulation of My-CaNHL4 protein also decreased. **g**–**i** Transient expression of *CaNHL4* decreases the infection of *P. syringae*. The disease development of *P. syringae* is presented, and the lesion size was measured at 2, 4, and 6 dpi. The expression of *HrpZ* gene was quantified by qPCR, revealing bacterial mass in the infected leaves at 4 dpi. The *HrpZ* gene expression levels were normalized to the expression of pepper actin. All experiments were repeated three times and showed similar results. The values represent the means ± standard errors (SEs) of three biological replications. The statistical analyses were performed using Student’s *t*-test (∗0.01 < *P* < 0.05; ∗∗0.001 < *P* < 0.01; ∗∗∗*P* < 0.001).

To further understand the function of CaNHL4 in defense against different pathogens, we generated a pART27-N-7::Myc-CaNHL4 expression vector and transiently overexpressed it in the leaves of pepper (*C. annuum*) by agroinfiltration, with a Myc:00 empty vector serving as the mock. At 2 dpi, western blotting was used to detect the accumulation of Myc-CaNHL4 protein. As shown in Fig. 7a, Myc-CaNHL4 proteins were detected in the infiltrated leaves. We inoculated *P. capsici*, *P. syringae*, and TMV on the infiltrated leaves at 2 d after infiltration. The disease development on the inoculated leaves was monitored and visualized via trypan blue staining. Figure 7b, c shows that overexpression of *Myc:CaNHL4* attenuated the disease development of *P. capsici*, as evidenced by reduced lesion size after trypan blue staining. Similarly, limited GFP signals were detected in the leaves overexpressing *CaNHL4* compared to mock leaves overexpressing the Myc:00 empty vector at 4, 5, and 6 dpi after TMV:GFP inoculation (Fig. 7d). The number of TMV CP transcripts in the leaves overexpressing *CaNHL4* was significantly lower than that in the mock plants at 4 and 6 dpi (Fig. 7e). Western blot analysis showed that the accumulation of TMV:GFP at 4 dpi was lower than that of the mock plants (Fig. 7f), indicating that overexpression of *CaNHL4* compromises the replication of TMV:GFP. After *P. syrinage* inoculation, the disease lesions were observed at 2, 4, and 6 dpi. Figure 7g, h show that the disease development of *P. syringae* on *CaNHL4*-overexpressing leaves was significantly slower than that on the mock leaves. Furthermore, we performed qPCR to quantify the expression of *HrpZ*, a pathogenic gene from *P. syringae*, to confirm the growth of *P. syringae*. As shown in Fig. 7i, at 4 dpi, the expression level of *HrpZ* in the *CaNHL4*-overexpressing leaves was significantly lower than that in the mock leaves, indicating that transient overexpression of *CaNHL4* reduces the infection of *P. syringae*. Taken together, the results show that silencing *CaNHL4* increases the susceptibility of pepper to these three pathogens but that transient overexpression of *CaNHL4* improves the resistance of pepper to these three pathogens, suggesting that CaNHL4 contributes to the resistance of pepper against different pathogens.

### CaNHL4 localizes to the plasma membrane

**Fig. 8:**
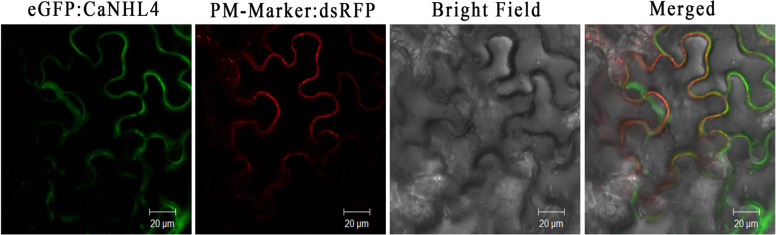
CaNHL4 localizes to the plasma membrane. Coexpression of *CaNHL4*:mGFP and the plasma membrane marker-dsRFP in the epidermal cells of *N. benthamiana* leaves after agroinfiltration. GFP and RFP fluorescence signals were visualized using confocal imaging at 48h after infiltration and are depicted in green and red, respectively. CaNHL4 was clearly visualized in the plasma membrane, as evidenced by the colocalization with the plasma membrane marker. The white scale bars represent 20μm.

To explore the subcellular localization of CaNHL4, we generated a CaNHL4:mGFP construct encoding a CaNHL4 protein fused to mGFP. The plasma membrane marker dsRFP was used as a positive control. *Agrobacterium tumefaciens* carrying either *CaNHL4:mGFP* or *PM-Marker-dsRFP* was coinfiltrated into the leaves of *Nicotiana benthamiana* plants. Two days after infiltration, green fluorescent signals were visualized using confocal microscopy, and CaNHL4-mGFP was found to be localized in the plasma membrane as it colocalized with the red fluorescent signals of the plasma membrane marker PM-Marker-dsRFP (Fig. 8).

### *CaNHL4* participates in the antistress defense of pepper by mediating the SA and JA signaling pathways and ROS production

**Fig. 9:**
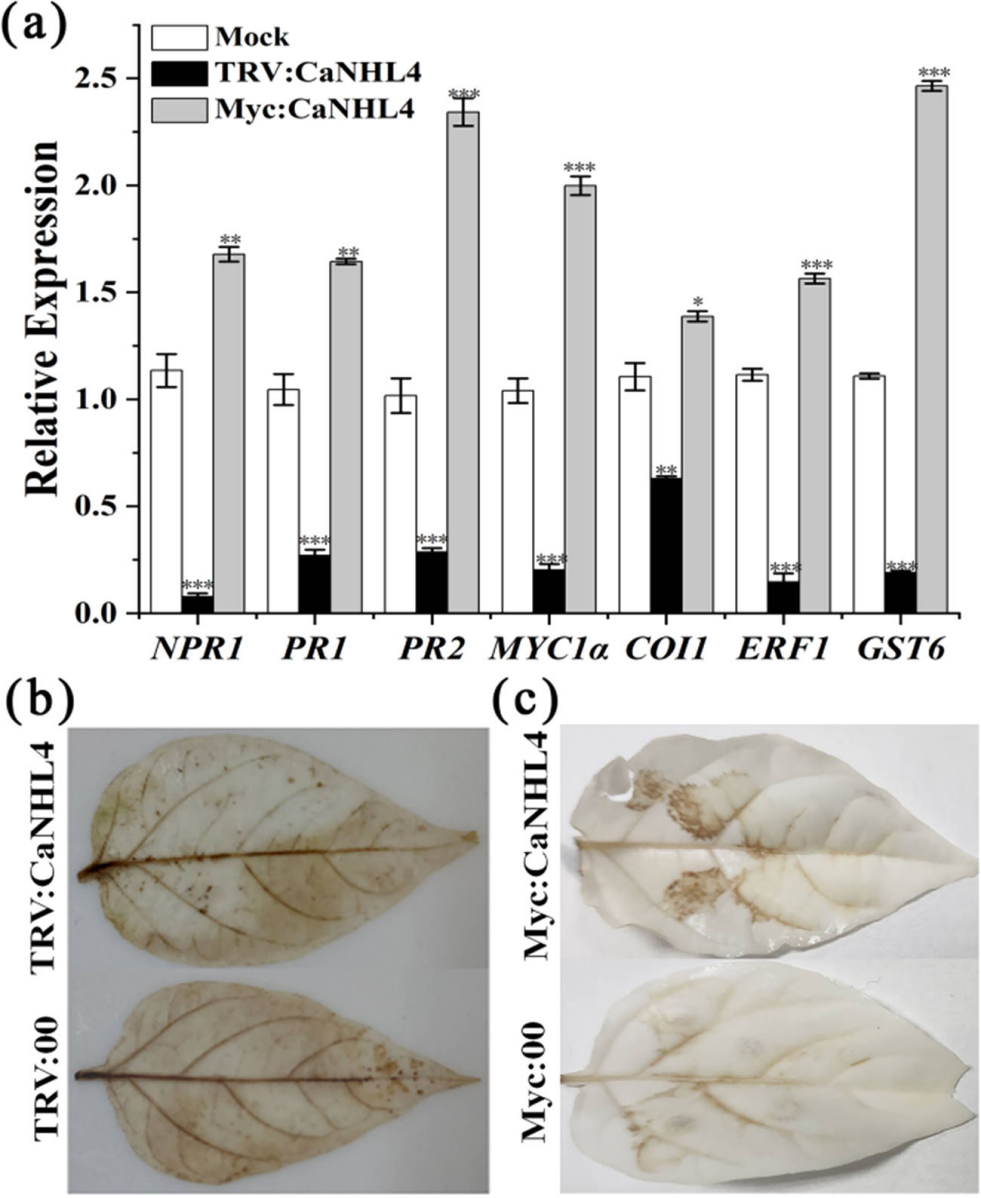
Silencing CaNHL4 reduces the expression of SA-responsive, JA/ET-responsive and ROS-responsive genes, and ROS production, whereas transient overexpression of CaNHL4 yields the opposite effects. **a** Effects of silencing and transient overexpression of *CaNHL4* on the expression of the SA-responsive genes *NPR1*, *PR1*, and *PR2*; the JA/ET-related genes *MYC1α*, *COI1*, and *ERF1*; and the ROS-induced gene *GST6*. **b** Effects of silencing *CaNHL4* on ROS production. **c** Effects of transiently overexpressing *CaNHL4* on ROS production. DAB staining was used to detect the ROS production. All the experiments were repeated three times and showed similar results. The values represent the means ± standard errors (SEs) of three biological replications. The statistical analyses were performed using Student’s *t*-test (∗0.01 < *P* < 0.05, ∗∗0.001 <*P* <0.01, ∗∗∗*P* < 0.001).

To further determine the effects of *CaNHL4* on the SA and JA signaling pathways, we examined the expression of SA-responsive genes, including non-expresser of pathogenesis-related gene 1 (*NPR1*), pathogenesis-related gene 1 (*PR1*), pathogenesis-related gene 2 (*PR2*)^19^, JA-responsive gene *MYC1α*, JA receptor coronatine insensitive 1 (*COI1*), and ethylene response factor 1 (*ERF1*)^20,21^, in *CaNHL4*-silenced leaves and CaNHL4-overexpressing leaves. Surprisingly, we found that the expression of these genes in the silenced plants decreased significantly (Fig. 9a), suggesting that silencing *CaNHL4* compromises the expression of these genes, resulting in the deactivation of the SA and JA signaling pathways. Similarly, we found that the expression level of the ROS-induced gene glutathione S-transferase 6 (*GST6*)^22^ was significantly decreased and that ROS production was significantly reduced in the silenced plants compared to that in the mock plants, as revealed by DAB staining, suggesting that *CaNHL4* is also involved in ROS production (Fig. 9a, b). However, overexpressing *CaNHL4* significantly increased the expression of SA-responsive and JA-responsive genes and the ROS-induced gene *GST6* (Fig. 9a), and increased ROS production was observed (Fig. 9c). In conclusion, our results indicated that *CaNHL4* affects the expression of SA-responsive and JA-responsive genes and ROS production, suggesting that *CaNHL4* might be involved in the antistress defense response of pepper by activating the SA and JA signaling pathways and ROS production.

## Discussion

It has been documented that *NHL* genes are involved in the process of plant disease resistance and stress tolerance in *Arabidopsis*, tobacco, soybean, grape, tomato, and other plant species^23^. In our study, we performed genome-wide identification of NHL genes in pepper. Functional analysis of the *CaNHL* genes revealed that *CaNHL4* plays an important role in the disease resistance of pepper.

On the basis of bioinformatic analysis, our results showed that members of CaNHL family contain the conserved LEA-2 domain. Previous studies have shown that members of the LEA protein family are involved in antistress responses^24–26^. For example, the gene encoding LEA-containing proteins is highly expressed in the late embryonic development of seeds and is highly expressed under environmental stresses, such as drought and low temperature^24^. Zegzouti et al. reported that the LEA-like protein ER5 is expressed in response to drought, ABA, and injury^25^. Singh et al. found that the expression of LEA14 in *A. thaliana* is induced under stress conditions, such as drought, coldness, osmotic stress, and high temperature^26^. Our findings revealed that some *CaNHL* genes encoding LEA-containing proteins are induced under stress conditions, such as cold, high temperature, ABA, JA, and SA treatments (Fig. 3), which is consistent with the results of previous studies. These findings suggest that members of the CaNHL protein family may be involved in the disease resistance of pepper.

Regulatory element analysis showed that the promoter regions of *CaNHL* genes contains many stress-responsive and defense-responsive-related elements, such as those involving MeJA, SA, and the drought response (Fig. 2a), suggesting that *CaNHL*s might participate in the pepper defense response. Many studies have shown that NHLs participate in the JA-mediated and ET-mediated defense response pathways^27^. In pepper, *CaNHL9* and *CaNHL10* contain 10 MeJA-responsive elements, *CaNHL4* has six MeJA-responsive elements, and the remaining genes, including *CaNHL11* and *CaNHL15*, also have MeJA-responsive elements (Fig. 2a). These findings suggest that some *CaNHL* genes might play a role in disease resistance by mediating the JA-signaling pathway. However, cis-acting elements of SA also are present within the promoter of *CaNHL4*, indicating that this gene might also participate in the SA-responsive signaling pathway. RNA-Seq and qPCR analysis showed that *CaNHL* genes are highly induced after exogenous MeJA and MeSA treatments (Figs. 3 and 4). In addition, we found that the expression of the JA/ET-responsive genes *MYC1α*, *COI1*, and *ERF1* and the SA-responsive genes *NPR1*, *PR1*, and *PR2* was also inhibited in *CaNHL4-*silenced plants (Fig. 9), providing strong evidence that *CaNHL4* is involved in stress resistance by mediating the JA and SA signaling pathways. Interestingly, we also found that many ABA-responsive elements are present in the promoter regions of *CaNHL* genes (Fig. 2a). Previous studies have shown that the gene encoding LEA-containing proteins is also induced in response to ABA^25^. In addition, ABA treatment of *A. thaliana* can upregulate SA-induced sesquiterpene synthesis, which indirectly indicates that ABA may participate in SA-mediated defense^28^. ABA is able to induce the expression of defense-related genes^29^, especially those that enhance plant resistance to viruses^30,31^. However, the role of ABA-responsive elements present in the promoter regions of *CaNHL*s remains unclear. We also found that the promoter region of each *CaNHL* gene contains light-responsive elements (Fig. 2), so we speculated that *CaNHL* genes might be induced by light. Our findings suggested that the activation of some *CaNHL* genes is not strongly correlated with the predicted cis-elements in the promoter region (Supporting Information 2). For example, we found that the promoters of both *CaNHL4* and *CaNHL6* contain SA-responsive elements. Thus, we hypothesized that *CaNHL4* and *CaNHL6* could be induced by SA treatment, which is in agreement with our findings that both *CaNHL4* and *CaNHL6* are induced by SA treatment (Fig. 4). Conversely, the promoter regions of *CaNHL2*, *CaNHL4*, *CaNHL6*, *CaNHL8*, *CaNHL10*, *CaNHL11*, and *CaNHL15* contain MeJA-responsive elements (Fig. 2a), indicating that these genes might be induced in response to MeJA. However, different expression patterns of these genes were observed on the basis of the RNA-seq data and qPCR analysis (Figs. 3 and 4). We speculated that the differences in their response to MeJA observed in our study might be due to the different number of MeJA-responsive elements in the promoters of individual *CaNHL* genes. ROS have been reported to act as antimicrobial agents and cross-links within the plant cell wall to block pathogen entry or to serve as local and systemic secondary messengers to trigger immune responses, such as the expression of defense-related genes or stomatal closure^32–35^. Xu et al. reported that UV-primed strawberry leaves induced disease resistance involving ROS production^36^. Similarly, we found that CaNHL4 is able to induce the production of ROS, resulting in a defense response against pathogen infection (Fig. 9).

It has been documented that JA and SA function as endogenous growth regulators that induce systemic resistance in plants^37^. Peng et al. treated *N. benthamiana* leaves with exogenous MeJA and found that the expression of *NbHIN1* was induced at 5 h after treatment, suggesting that *NbHIN1* may be involved in the JA pathway^38^. In this study, we found that the expression levels of *CaNHL1*, *CaNHL4*, *CaNHL6*, *CaNHL10*, *CaNHL11*, and *CaNHL12* significantly increased after exogenous MeSA treatment (Fig. 5), indicating that CaNHL may also be involved in the response of pepper to SA. Furthermore, our results showed that the *CaNHL1*, *CaNHL4*, and *CaNHL12* genes are highly induced in response to the infection by *P. capsici*, *P. syringae*, and TMV (Fig. 6), which further confirms that these genes might be involved in the biotic stress response of pepper. Interestingly, we found that the *CaNHL* genes significantly induced in response to abiotic and biotic stresses clustered into the same group (I or III) based on the phylogenetic tree analysis (Fig. 1). These findings suggested that NHL family members in group I and group III may be involved in plant defense. Therefore, we speculate that the *NHL* genes of *S. lycopersicum* and *N. tabacum* in this subgroup may also participate in disease resistance.

Numerous studies have shown that NHL proteins in the NHL family localize to the plasma membrane^38–40^. Similarly, the present localization study of CaNHL4 revealed its localization to the plasma membrane (Fig. 8). Studies on the disease resistance protein AtNDR1 have shown that the membrane-localized AtNDR1 protein tends to accumulate in the cell membrane near the infection site, which facilitates quick sensing of pathogens, thus resulting in rapid activation of the defense response^39,41^. CaNHL4 localizes to the plasma membrane, and it is speculated that CaNHL4 may also be involved in sensing pathogens and quickly inducing a defense response. Our experiments revealed that the silencing of *CaNHL4* enhances the infection of *P. capsici*, *P. syringae*, and TMV, suggesting that *CaNHL4* is involved in the defense response of pepper to these three pathogens (Fig. 6). A growing body of evidence has shown that NHL family proteins are involved in plant resistance against various pathogens. For example, the expression of *OsHIN1* in rice increases blast resistance^42^, and overexpression of *AtNHL3* in *A. thaliana* significantly improves resistance to *Pseudomonas*^39^. Taken together, these findings suggest that *CaNHL4* is involved in the rapid disease resistance of plants to pathogens.

Our findings presented in this study highlight the importance of *CaNHL4* in the disease resistance of pepper. To the best of our knowledge, this is the first study to identify the *CaNHL* genes and examine their expression profiles in a genome-wide manner in pepper. Functional analysis showed that CaNHL is involved in JA-induced and SA-induced plant stress resistance and that CaNHL4 plays an important role in pepper stress resistance. In the future, gene editing, such as CRISPR/Cas9 and proteomics technologies, will be used to further understand the role of CaNHL4 in the JA and SA signaling pathways.

## Materials and methods

### Plant materials and bacterial strains

The pepper cultivar ‘Zunla-1’ (*Capsicum annuum* L.) and tobacco (*N. benthamiana*) were grown in the greenhouse under a 14 h/10 h light/dark photoperiod, 75% relative humidity and 25 °C.

*A. tumefaciens* GV3101 was grown in LB media supplemented with kanamycin and Rif at 28 °C in an orbital shaker at 200 rpm and harvested during the log phase of growth for infiltration.

### Identification of CaNHL family genes

By using the BLASTp program, we searched the amino acid sequence of the HIN1 protein in the *C. annuum* Zunla-1 whole genome database of the Sol Genomics Network^43,44^ (https://solgenomics.net/) and selected the sequences with *E* ≤ 1e−10 as candidate sequences. ProtParam tool (https://web.expasy.org/protparam/) was used to calculate the MW, pI, and chemical formula. We then used SMART (http://smart.embl-heidelberg.de/) to predict the domain architectures of these proteins, and those containing NHL LEA-2 conserved domains were identified as CaNHL family genes.

### Bioinformatic analysis

A phylogenetic tree was constructed using the amino acid sequences translated from the coding sequences of the *CaNHL* genes together with the amino acid sequences of SlNHL of *S. lycopersicum* and NtHIN1 of *N. tabacum*. Based on the neighbor-joining (NJ) method, a phylogenetic tree was constructed by the MEGA 7.0.26 program. The bootstrap value was set to 1000, and the Poisson model was used, allowing an estimation of the confidence of the results of the phylogenetic tree.

For regulatory element analysis, we extracted 1500 bp upstream sequences of thee above-mentioned genes from the genome. The PlantCare database (http://bioinformatics.psb.ugent.be/webtools/plantcare/html/) was used to predict the regulatory element components^45^, and TBtools was used to display the results^46^.

For motif identification, the multiple expectation maximization for motif elicitation (MEME) program (http://meme.nbcr.net/meme/intro.html) was used to identify the conserved motifs in the identified CaNHL proteins^47^. The optimization parameters were as follows: number of repetitions, any; maximum number of motifs, 15; and optimum width of each motif, between 6 and 50 residues. TBtools was used to show the motif structure^46^.

### RNA-Seq analysis

By analyzing the pepper transcriptomic data available in the Pepper Informatics Hub (http://pepperhub.hzau.edu.cn/)^48^, we determined the expression patterns of the members of the CaNHL family in different tissues, including the leaves, stems, roots, flowers, petals, stamens, and fruits, and the expression patterns under different stress treatments, including cold, heat, NaCl, SA, ABA, and JA. TBtools was used to visualize the results.

### Plant treatment with exogenous hormones

Pepper (*C. annuum*) plants at the six-leaf stage were sprayed with 0.1 mmol/L MeJA or MeSA (Sigma-Aldrich, USA). Mock plants were sprayed with sterile water. Samples were collected at 3-h intervals for up to 12 h, immediately frozen in liquid nitrogen and stored at −80 °C for RNA isolation.

### Pathogen inoculation and quantitative RT-PCR

For TMV inoculation, 100 μL extracts of TMV-GFP-infected leaves were applied to each leaf by rubbing, after which leaves were imaged under UV light at 2 and 4 d after inoculation. Each experiment was repeated three times, with at least three independent plants used each time. For *P. capsici*, we inoculated fresh fungal plugs for 3 d into fresh pepper leaves and removed the plug on the second day after inoculation. The lesion size was observed at 3 dpi. Trypan blue staining was used to visualize the lesions. For *P. syringae*, a fresh bacterial overnight culture was adjusted to OD600 of 0.8 with sterile water, and then injected into the pepper leaves by a syringe. The lesion sizes were monitored and measured at 2, 4, and 6 dpi.

Real-time quantitative PCR (qPCR) was carried out by a CFX Touch Real-Time PCR System (Bio-Rad, USA) and a QuantiNova SYBR Green PCR Kit (Qiagen, Germany) to determine the relative expression level of the target gene. Primer 5.0 software was used to design gene-specific primers according to the coding sequence of each gene. *UBI3* was used as the internal reference gene^49^, and the relative changes in gene transcription levels were measured by the 2^−ΔΔCT^ method.

### Vector construction

For VIGS, pTRV was used^50^. A 200 bp fragment of *CaNHL4* (MN961192) was selected through the VIGS tool (https://vigs.solgenomics.net/); this fragment is specific to *CaNHL4* and has no mismatches with other CaNHLs. The PCR-amplified fragment was cloned into a pTRV2 vector using the restriction sites of *Bam*HI and *Xho*I.

For subcellular localization, the open-reading frame of *CaNHL4* was cloned into a pART27-N-mGFP vector by using the restriction sites of *Eco*RI and *Xba*I.

For transient expression of *CaNHL4*, we digested the *CaNHL4* fragment using the restriction sites of *Eco*RI and *Xba*I from pART27-N-eGFP-CaNHL4 and cloned it into a pART27-N-7*Myc vector. All the primers used are provided in Supporting Information 3.

### Agroinfiltration and confocal microscopy

pART27:CaNHL4-N-mGFP, pART27-N-7*Myc-CaNHL4, and pTRV2:CaNHL4 were transformed into *Agrobacterium* strain GV3101. *Agrobacterium*-mediated transient expression was performed following the methods described previously^51^. For VIGS, *Agrobacterium* strain GV3101 carrying pTRV1, pTRV2:00, or pTRV2:CaNHL4 was adjusted to an OD600 of 0.4 and mixed at a 1:1 ratio, after which the mixture was infiltrated into the leaves of *C. annuum* plants at the four-leaf stage. For localization, infiltrated *N. benthamiana* leaves were harvested at 36 h after infiltration, and leaf discs were visualized using LSM780 confocal laser scanning microscope equipped with a ×40/1.2 water-immersion objective (Zeiss, Germany). Excitation of RFP was performed at 543 nm with a HeNe laser. A 590–620 nm filter was used to capture the emissions. Excitation of GFP was performed at 488 nm with an Ar-ion laser, and the emissions were captured with a 505–530 nm pass filter. Images were scanned eight times.

### Diaminobenzidine (DAB) staining

DAB (BBI Life Science, China) was used to detect ROS production at 2 d after inoculation, the leaves were treated with 1 mg/mL DAB solution for 6 h, after which the leaves were decolorized in the decolorizing solution, which was composed of ethanol:acetic acid:glycerol at a ratio of 3:1:1, at 95 °C for 15 min.

### Statistical analysis

All the experiments and data presented here involved at least three repeats. The data are presented as the means and standard deviations. The statistical analyses were performed with SPSS software (version 19.0) using Student’s *t*-test.

## Supplementary information

Supplemental Material
