# Supplementary material for: Genome-wide analysis of NDR1/HIN1-like genes in pepper (Capsicum annuum L.) and functional characterization of CaNHL4 under biotic and abiotic stresses
Source: Hortic Res. 2020 Jun 1;7:93. doi: 10.1038/s41438-020-0318-0 (PMC7261774; doi:10.1038/s41438-020-0318-0)
Supplement: Supplementary file 1 — Supplemental Material [file 41438_2020_318_MOESM1_ESM.docx]

Appendix

**Supporting information 1**. The predicted three-dimensional structures of the CaNHL proteins. The PHYRE2 (http://www.sbg.bio.ic.ac.uk/phyre2/html/page.cgi?id=index) was used to predict the three-dimensional structure of the CaNHL proteins. ExMol (http://www.sbg.bio.ic.ac.uk/~ezmol/) was used to display the protein. PHYRE2 showed the confidence of the models were all greater than 97.5%, except of CaNHL12, whose confidence was 25.5%. Images are rainbow coloured from the N terminus to C terminus. All CaNHL proteins except of CaNHL12 share similar protein structures.

**Supporting information 2**. Comparison of predicted regulatory elements and qPCR results. The number of SA- and MeJA-responsive cis-regulatory elements present in promoters of CaNHLs was compared with the expression of CaNHLs under MeJA and MeSA treatments.

**
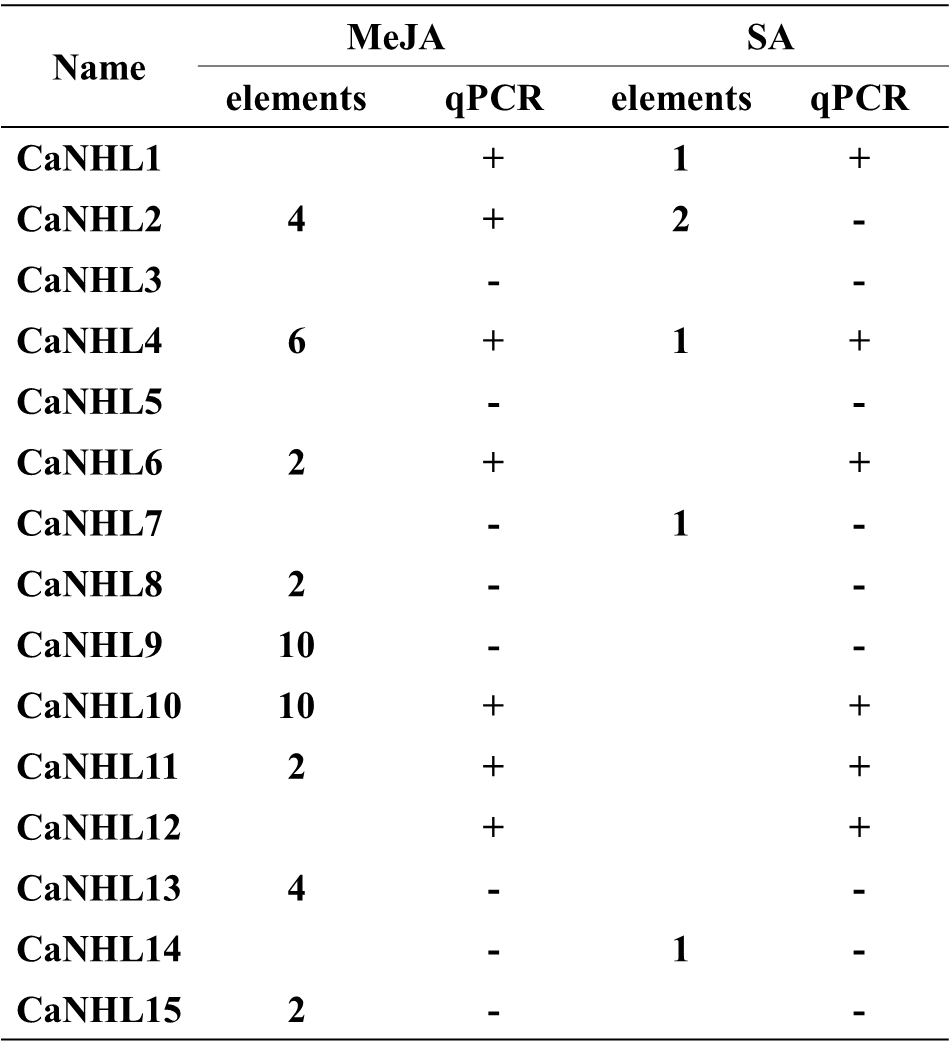
**

**Supporting information 3**. The primers used in this study.

| **Name** | **Nucleotide sequence(5`to 3`)** |
| --- | --- |
| qCaNHL1 | TCCGGCATTGACCTTTCTAC |
|  | AACGGCACTTTCCCTTTCTT |
| qCaNHL4 | GGTGCTTTAGCTGCTGCTTT |
|  | TTCTTGTTGGGGTTTCTTGC |
| qCaNHL6 | TGCAGTGAATCCCAACAAGA |
|  | TTGTGCTGCCTCTTGAATTG |
| qCaNHL10 | GTAGCCGTGGTGGTAGCAGT |
|  | TTGGCAGTGGACAAATCAAA |
| qCaNHL11 | CAGTTTGCATCCGGTGTTTA |
|  | TGTGCGTTTTAATCCAACCA |
| qCaNHL12 | TAGCAGTTTGCATCCAGTGC |
|  | GGACTCCAAAGGAACCTTGA |
| qCaUBI3 | GCCCTTATGCTGGAGGTGTA |
|  | GGACTCCACTGCTCCTTGAG |
| CaNHL4-mGFP | GGAATTCATGGCAGATCATCAAAGAATCCATCCAG |
|  | CGTCTAGATTAAAGCCTAAATCCAAACTTGC |
| TRV:CaNHL4 | GCTCTAGA ATGGCAGATCATCAAAGAATCCA |
|  | CGGGATCC TGGTTGGAAGACAAAGTAG |
| TRV CP-qPCR | TCATTTGACAAGTCGGGC |
|  | TGTGTTTGGATTCGCAGG |
| TMV CP-qPCR | TTAGGTTCCCTGACGGTGAC |
|  | ACCGTTGCGTCGTCTACTCT |
| qNPR1 | TGTTGCTGCCATGAGAAAAG |
|  | TAACCGATCCTTTGGAGCAG |
| qPR1 | CGTGAAGATGTGGGTCAATG |
|  | CCATACGGACGTTGTCCTCT |
| qPR2 | TCTTTTTGATGCCCTTTTGG |
|  | CGTCCAGGTTTCTTTGGTGT |
| qCOI1 | CAGCAGCCCATTGTTTCTTAC |
|  | TACTGGCCAAGTACTTCCAATC |
| qMYC1α | CGGTTCTTCTTCCGTCTTCTT |
|  | ACGCTGTTGAAGGGTTTCT |
| qERF1 | GCTCTTAACGTCGGATGGTC |
|  | AGCCAAACCCTAGCTCCATT |
| qGST6 | TCACAATGGAAAGCCCATTT |
|  | TTGCTCCTCTCCTTTTCCAA |
| qHrpZ | CCCAGGAGCTGACTCACAAT |
|  | GCACCTGTGTCATCAAGTCG |
